# Advances and challenges in the origin and evolution of ovarian cancer organoids

**DOI:** 10.3389/fonc.2024.1429141

**Published:** 2024-08-16

**Authors:** Mengpei Zhang, Rutie Yin, Kemin Li

**Affiliations:** ^1^ Department of Obstetrics and Gynecology, West China Second University Hospital of Sichuan University, Chengdu, China; ^2^ Key Laboratory of Birth Defects and Related Diseases of Women and Children (Sichuan University), Ministry of Education, Chengdu, China

**Keywords:** organoids, patients-derived organoids, ovarian cancer, advances and challenges, review

## Abstract

Despite advancements in cancer research, epithelial ovarian cancer remains a leading threat to women’s health with a low five-year survival rate of 48%. Prognosis for advanced cases, especially International Federation of Gynecology and Obstetrics (FIGO) III-IV, is poor. Standard care includes surgical resection and platinum-based chemo, but 70-80% face recurrence and chemoresistance. In recent years, three- dimensional (3D) cancer models, especially patients-derived organoids (PDOs), have revolutionized cancer research for personalized treatment. By transcending the constraints of conventional models, organoids accurately recapitulate crucial morphological, histological, and genetic characteristics of diseases, particularly in the context of ovarian cancer. The extensive potential of ovarian cancer organoids is explored, spanning from foundational theories to cutting-edge applications. As potent preclinical models, organoids offer invaluable tools for predicting patient treatment responses and guiding the development of personalized therapeutic strategies. Furthermore, in the arena of drug evaluation, organoids demonstrate their unique versatility as platforms, enabling comprehensive testing of innovative drug combinations and novel candidates, thereby pioneering new avenues in pharmaceutical research. Notably, organoids mimic the dynamic progression of ovarian cancer, from inception to systemic dissemination, shedding light on intricate and subtle disease mechanisms, and providing crucial insights. Operating at an individualized level, organoids also unravel the complex mechanisms underlying drug resistance, presenting strategic opportunities for the development of effective treatment strategies. This review summarizes the emerging role of ovarian cancer organoids, meticulously cultivated cellular clusters within three-dimensional models, as a groundbreaking paradigm in research.

## 1 Introduction

In recent years, despite remarkable advancements in cancer research and therapeutic strategies, epithelial ovarian cancer remains a significant threat to women’s health, with a dismal five-year survival rate of just 48% (1). The prognosis for patients diagnosed with advanced ovarian cancer, particularly those in FIGO stages III-IV, is often bleak. Currently, the standard of care involves surgical resection followed by platinum-based chemotherapy. However, a significant proportion of patients, approximately 70% to 80%, face the daunting challenges of tumor recurrence and increasing chemoresistance following initial treatment (2). In recent decades, the emergence of novel targeted drugs has revolutionized the treatment paradigm for ovarian cancer. PARP inhibitors, which disrupt DNA repair mechanisms, leading to cancer cell death, have gained widespread acceptance in the maintenance therapy of advanced ovarian cancer. These inhibitors have demonstrated remarkable efficacy in prolonging PFS among patients with advanced disease, revolutionizing the treatment landscape. Nevertheless, due to the inherent heterogeneity and polymorphism of ovarian cancer, the overall response rate to these agents remains around 20% (3). The development of advanced, recurrent, or chemoresistant disease is not the sole contributor to poor clinical outcomes in ovarian cancer patients. The extensive intraperitoneal dissemination of lesions also poses a significant challenge. Tumor cells detached from the primary tumor can disseminate to the peritoneum and omentum, and even disseminate throughout the abdominal cavity via ascites, a common occurrence in advanced ovarian cancer (4). Central to this complex process of metastasis and recurrence are ovarian cancer stem cells. These cells possess the ability to self-renew and initiate tumorigenesis, while exhibiting inherent resistance to cytotoxic therapies. Consequently, they often survive the rigors of standard chemotherapy and drive the regrowth and recurrence of the tumor (5).Given the intricate nature of this pathological process, clinicians often encounter limitations in relying solely on clinical guidelines and past experiences when managing patients with advanced, recurrent, or refractory ovarian cancer. Selecting the most appropriate drugs and achieving personalized precision treatment can be a daunting task. Therefore, the development of a preclinical model capable of accurately predicting the treatment response of ovarian cancer and guiding individualized treatment strategies has been a priority in scientific research.

In recent years, three-dimensional (3D) cancer models, especially patients-derived organoids (PDOs), have revolutionized cancer research for personalized treatment. By transcending the constraints of conventional models, organoids accurately recapitulate crucial morphological, histological, and genetic characteristics of diseases, particularly in the context of ovarian cancer. The extensive potential of ovarian cancer organoids is explored, spanning from foundational theories to cutting-edge applications. As potent preclinical models, organoids offer invaluable tools for predicting patient treatment responses and guiding the development of personalized therapeutic strategies (6). Furthermore, in the arena of drug evaluation, organoids demonstrate their unique versatility as platforms, enabling comprehensive testing of innovative drug combinations and novel candidates, thereby pioneering new avenues in pharmaceutical research. Notably, organoids mimic the dynamic progression of ovarian cancer, from inception to systemic dissemination, shedding light on intricate and subtle disease mechanisms, and providing crucial insights. Operating at an individualized level, organoids also unravel the complex mechanisms underlying drug resistance, presenting strategic opportunities for the development of effective treatment strategies (7). Furthermore, in the arena of drug evaluation, organoids demonstrate their unique versatility as platforms, enabling comprehensive testing of innovative drug combinations and novel candidates, thereby pioneering new avenues in pharmaceutical research. Notably, organoids mimic the dynamic progression of ovarian cancer, from inception to systemic dissemination, shedding light on intricate and subtle disease mechanisms, and providing crucial insights. Operating at an individualized level, organoids also unravel the complex mechanisms underlying drug resistance, presenting strategic opportunities for the development of effective treatment strategies. This review summarizes the emerging role of ovarian cancer organoids, meticulously cultivated cellular clusters within three-dimensional models, as a groundbreaking paradigm in research.

## 2 Trends in ovarian cancer organoid research

**Figure 1:**
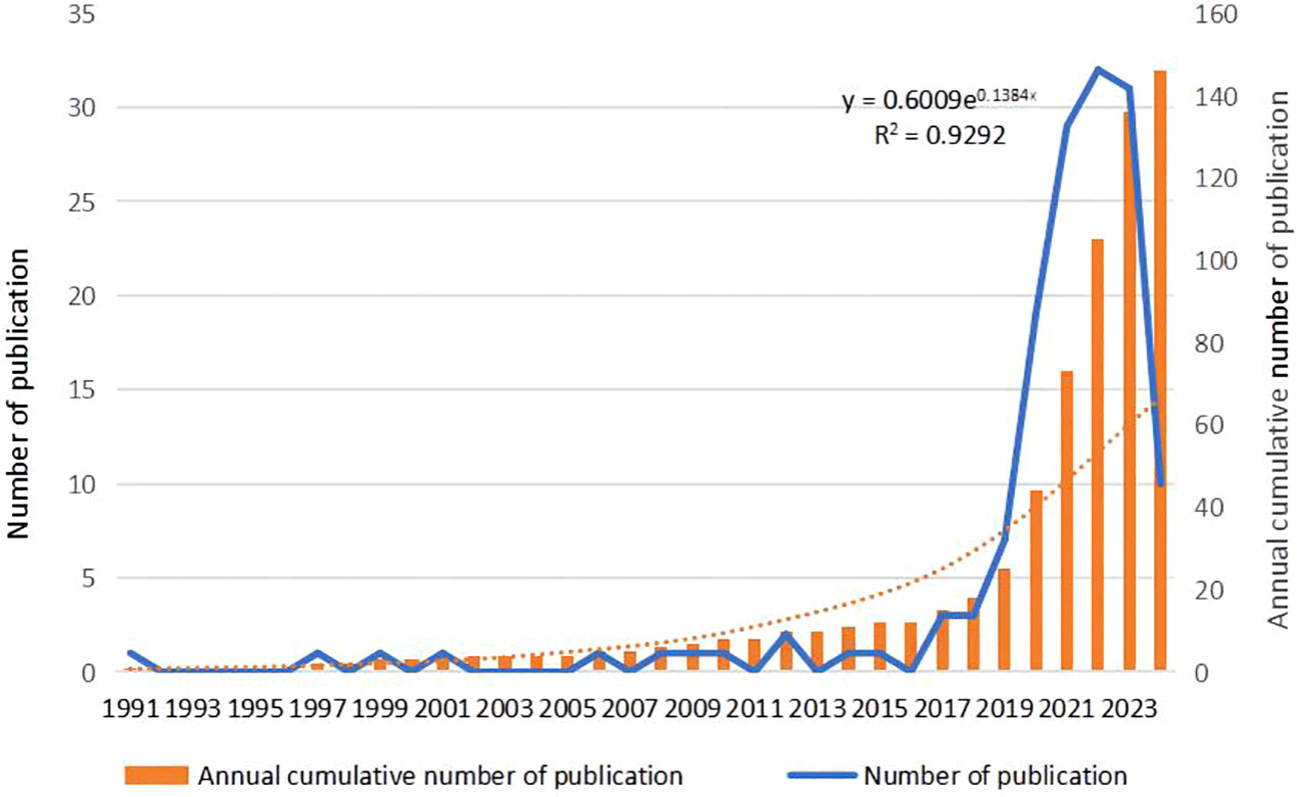
Annual frequency of publications in the field of ovarian cancer organoids. The orange bar chart represents the annual cumulative number of publications, the blue line represents the annual number of publications, and the orange dashed line represents the polynomial fitting curve.

**Figure 2:**
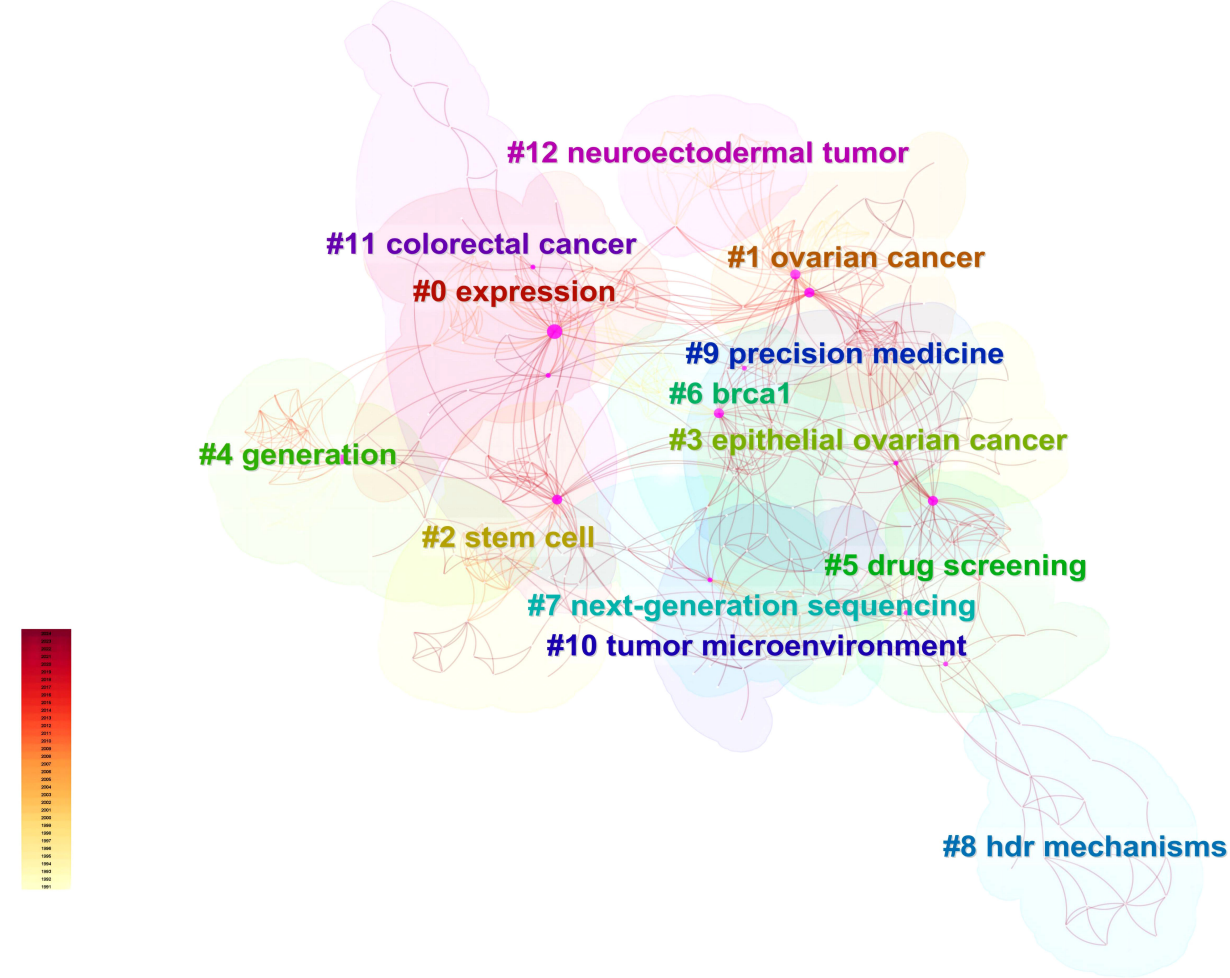
Clusters of the keywords. Different colors represent different clusters of keywords.

**Figure 3:**
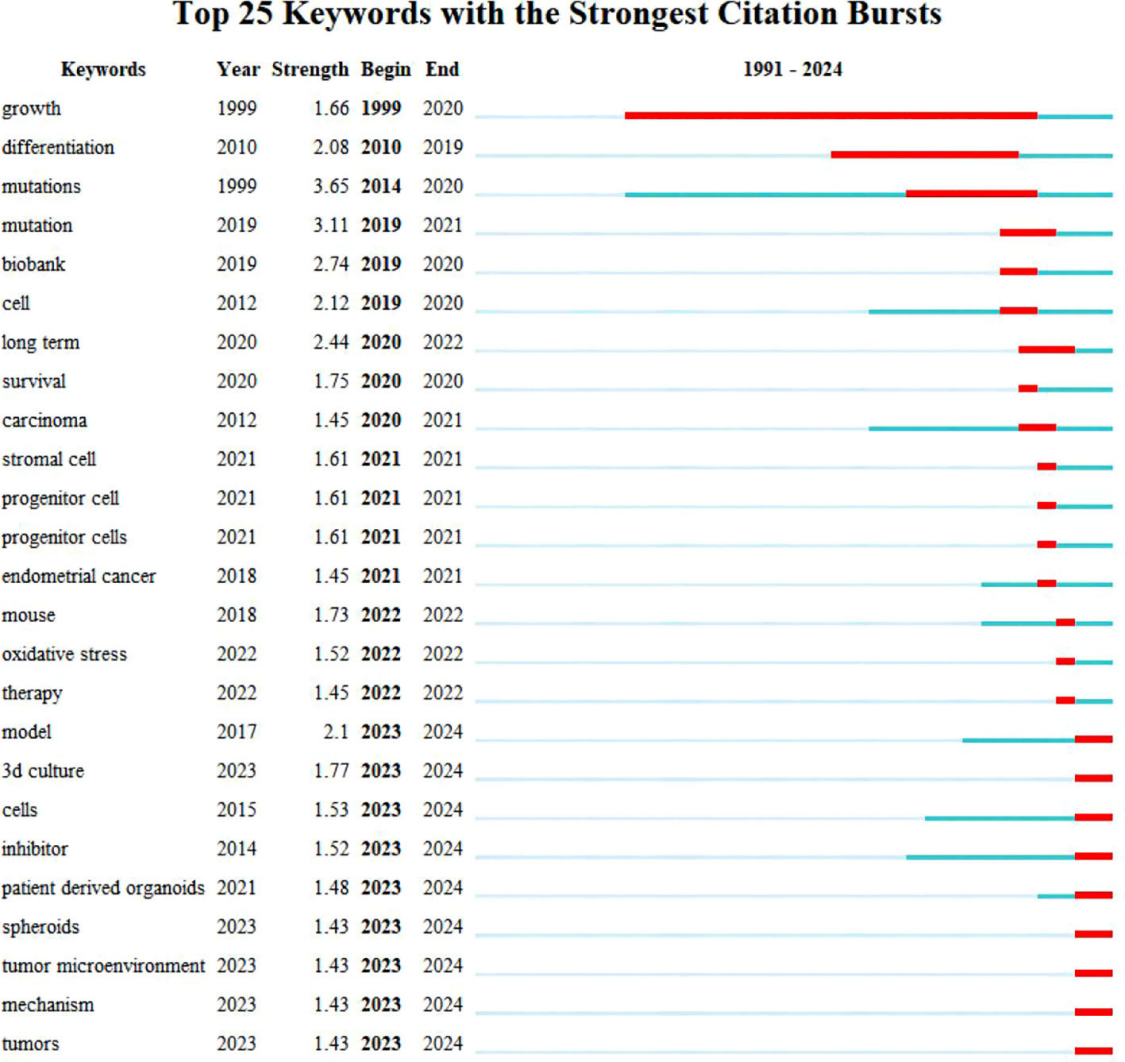
Top 25 keywords with the Strongest Citation Bursts. Blue bars and red bars mean that some keywords are cited frequently in a certain period.

Utilizing Web of Science, a widely recognized academic resource platform, we conducted a comprehensive search using “ovarian cancer” and “organoid” as core keywords. After a rigorous process of deduplication and screening, we carefully selected 146 high-quality articles for analysis. Subsequently, we employed the advanced literature analysis tool CiteSpace to delve deeply into these articles. Through a meticulous examination of the trend in annual publication volume, we observed a consistent increase in the popularity of ovarian cancer organoid research since 2012, stabilizing at a relatively high level from 2021 to 2023, with approximately 30 relevant articles published annually (**Figure 1**). This trend clearly demonstrates the activity and attention in this field of research. In the keyword clustering analysis, we further identified multiple research hotspots in the field of ovarian cancer organoid research. These hotspots encompassed ovarian cancer tumor stem cells, organoid passaging, studies on disease pathogenesis, drug screening, and precision medicine (**Figure 2**). The clustering of these keywords not only reflects the current focal directions of research but also suggests potential future trends. Additionally, the emergence analysis of literature keywords provided a visual representation of significant advancements in ovarian cancer organoid research in recent years. Since 1999, organoid culture techniques have gradually garnered widespread attention and have made notable progress in the past two decades. Nowadays, the culture of ovarian cancer organoids has become relatively mature, providing researchers with a more reliable and effective experimental model. Recently, the utilization of tumor organoids for the study of tumor microenvironments and tumorigenesis mechanisms has emerged as a new research hotspot (**Figure 3**). This trend undoubtedly furthers the deepening development of ovarian cancer organoid research. These comprehensive analytical results visually present the booming development of ovarian cancer organoid research. With continuous technological advancements and deepening research, we have reason to believe that this field will achieve more breakthroughs and outcomes in the future.

## 3 Generation and development of ovarian cancer organoid models

The ovarian surface is distinctively composed of a single layer of flat or cuboidal epithelial cells, supported beneath by a thin layer of dense connective tissue referred to as the tunica albuginea. The ovarian parenchyma is further divided into an outer cortex and a central medulla. The genesis of ovarian cancer is multifaceted, stemming from various cell types within the ovary. Ovarian carcinoma is distinguished by its intricate heterogeneity, manifesting at molecular, cellular, and anatomical levels. This multifaceted heterogeneity profoundly influences the responsiveness to surgical interventions and/or systemic therapeutic strategies, concurrently fostering both intrinsic and acquired drug resistance mechanisms. Epithelial ovarian cancer, which comprises the majority of cases, encompasses various histological subtypes that have been grouped into two broad categories based on histopathological, molecular, and genetic criteria. Type I tumors, which include low-grade serous carcinoma (LGSOC), mucinous, endometrioid, clear cell, and transitional carcinomas, are known for their indolent growth and comparatively favorable prognosis. In contrast, Type II tumors, notably high-grade serous ovarian carcinoma (HGSOC), undifferentiated carcinoma, and carcinosarcoma, exhibit a more aggressive clinical course (8). HGSOC, accounting for approximately 70% of OC cases, is particularly devastating, with a 5-year survival rate of merely 30% (1). Historically, epithelial ovarian cancer was postulated to originate from the ovarian surface epithelium. However, with the evolution of research, particularly in molecular biology and genetics, scientists have postulated a hypothesis suggesting that high-grade serous carcinoma might originate from the epithelium of the fallopian tube (9). The standard treatment for HGSOC is surgical cytoreduction, sequentially followed by platinum-based chemotherapy. However, 70% of patients relapse within 2 years, and most recurrent cases develop chemoresistance, becoming unresponsive to standard therapies (10).

The suboptimal clinical outcomes experienced by HGSOC patients are multifaceted, encompassing not only the recurrence of chemoresistant disease but also the extensive dissemination of the malignancy within the peritoneal cavity. Cancerous cells detach from primary tumors and have the potential to migrate to the peritoneum and omentum, which are primary sites for HGSOC metastasis (11). These cells then propagate throughout the abdominal cavity, utilizing peritoneal fluid or ascites (a common occurrence in advanced OC patients) as vehicles for dissemination. At the heart of this metastatic and recurrent cycle lie ovarian cancer stem cells (OCSCs), alternatively known as cancer-initiating cells (CICs), which act as pivotal drivers. Inherently, OCSCs possess the capacity for self-renewal and the ability to initiate tumorigenesis, coupled with a natural resilience against cytotoxic therapies. Consequently, they evade the effects of standard chemotherapy, contributing to tumor regrowth and relapse (10).

Recognizing the intricacies of this scenario, there is a pressing demand for preclinical models that can holistically replicate the diverse characteristics of EOC and facilitate efficient drug discovery efforts. Given the limitations of traditional immortalized cancer cell lines in accurately mirroring the behavior of this heterogeneous disease, recent years have witnessed a surge in interest towards models derived from patient materials. These models offer promising avenues for addressing the complexities of HGSOC, from tumor heterogeneity to the intricate interplay between tumor cells and their microenvironment, while also advancing personalized medicine and drug discovery strategies.

### 3.1 Generation of ovarian cancer organoids

**Figure 4:**
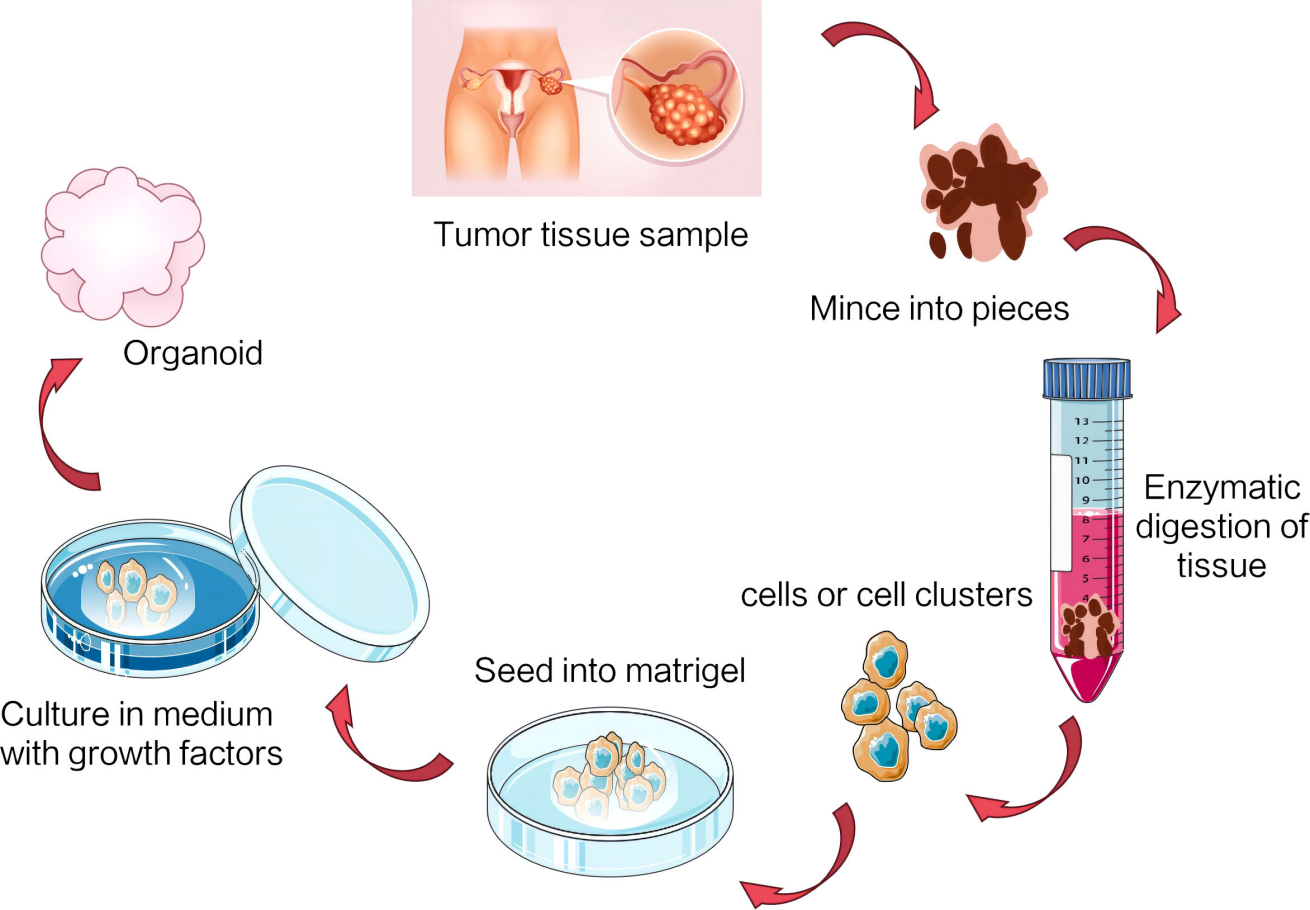
The common methods and processes for establishing ovarian cancer organoids.

Currently available for ovarian cancer research are a range of preclinical models, including two-dimensional (2D) cell lines, patient-derived xenograft (PDX) models, and organoids. 2D cell lines have historically been the mainstay of cancer research, particularly for initial discoveries and drug screening. However, their limited capacity to faithfully recapitulate the complexity of patient tumor behavior and molecular phenotypes has led to concerns over clinical reproducibility (12). On the other hand, PDX models offer a more accurate simulation of the original tumor characteristics. Nevertheless, their establishment is a complex, costly, and time-consuming process, often raising ethical concerns (13). In an effort to address these limitations, researchers are actively optimizing existing models and exploring innovative technologies such as three-dimensional (3D) cell culture. This approach aims to more accurately mimic tumor growth and behavior, thereby propelling progress in cancer research. Organoids, a sophisticated 3D cell culture system, stand at the forefront of this endeavor. They recapitulate the structural and functional intricacies of real biological organs. Leveraging stem cell technology, specifically adult stem cells or pluripotent stem cells, organoids are induced to differentiate under specific culture conditions, ultimately self-assembling into 3D structures (**Figure 4**). With meticulous design and regulation, organoids can closely resemble the cell types, organ structures, and physiological functions of real organs (14). As a pioneering biomedical technology, organoids have garnered significant attention in cancer research in recent years, offering a promising avenue for deeper insights into ovarian cancer and potentially revolutionizing therapeutic strategies.

In the realm of ovarian cancer research, multiple teams have successfully developed ovarian cancer organoids (**Table 1**), significantly enhancing our understanding of the disease’s biological characteristics and enabling the exploration of novel therapeutic strategies. In 2018, Hill et al. (15)made a groundbreaking achievement by utilizing ovarian tumor tissues and pleural effusion from 22 ovarian cancer patients to create tumor cell particles, resulting in the successful cultivation of 33 high-grade serous ovarian cancer organoid models with a near-perfect culture success rate. Remarkably, these organoids retained the characteristics of the original tumor, and genetic sequencing confirmed a high degree of consistency with ovarian tumor tissues, establishing their potential as predictive tools for clinical responses (15). Building on this progress, the esteemed Hans Clevers research group in the field of organoids made significant contributions in 2019. They established an organoid culture system utilizing tissues from ovarian cancer patients, successfully cultivating 56 organoid strains from tissues of 32 different patients. This effort culminated in the creation of the first live ovarian cancer biobank, encompassing a diverse array of epithelial ovarian cancer subtypes. Notably, their findings demonstrated that these organoids maintained their original tumor’s histological structure, biomarker expression, and genomic characteristics even after multiple passages, faithfully replicating the overall genomic landscape of ovarian cancer in terms of copy number variations and common mutations (16).

**Table 1:** PDO platform establishment in ovarian cancer research.

Author (year)	Number of organoid lines	Types of organoid lines	Sample source	Representative Validation
Hill et al. (15)	33	HGS	22 HGSOC tissues and Pleural puncture fluid	H&E staining; IHC; WES
Kopper et al. (16)	56	all main subtypes of OC	13HGSOC tissues; 5 LGSOC tissues; 3 SBT tissues; 2 MA tissues; 5 MBT tissues; 1 OCCC tissue; 1 ENOC tissue	H&E staining; IHC; GS; RNA sequencing; genetic manipulation; drug screening
Hoffmann et al. (17)	15	HGS	13 HGSOC primary tumor deposits	WGS; Mutational analysis
Maenhoudt et al. (20)	13	main subtypes of OC	22 HGSOC tissues; 2 LGSOC tissues; 1 MC tissue; 1 OCCC tissue; 1 malignant mixed mesonephric tumor tissue	H&E staining; IHC; immunofluorescent analysis; WES; low-coverage WGS; qRTPCR; drug sensitivity testing
Zhang et al. (18)	many	HGS	fallopian tubes	WGS; drug sensitivity testing; secretion factor analysis; transcriptome sequencing.
Ding (46)	10 ovarian cancer 3D organoids; 2 ovarian cancer ALI organoids	HGS;CCC;SBT	34 ovarian cancer tissues and fresh blood	WGS; CNVs; SNVs; SVs
Senkowski et al. (19)	17	HGS	10 HGSOC tissues	WGS; IHC; single-cell RNA sequencing

HGS, high-grade serous; HGSOC, high-grade serous ovarian cancer; LGSOC, low-grade serous ovarian cancer; SBT, serous borderline tumor; MA, mucinous adenocarcinoma; MBT, mucinous borderline tumor; MC, mucinous cystadenocarcinoma; OCCC, ovarian clear cell carcinoma; H&E, hematoxylin and eosin; IHC, immunohistochemistry; WES, whole-exome sequencing; WGS, whole-genome sequencing.

Ovarian cancer organoids are primarily derived from fresh surgical specimens, which have a short survival time, require proximity between hospitals and research institutions, and involve expensive infrastructure support. These factors greatly limit the establishment of ovarian cancer organoids and their widespread application in ovarian cancer research. However, recently, Wojciech Senkowski and his colleagues successfully developed two medium formulations that can be used for long-term culture and expansion of HGSC organoids. Using this method, they successfully cultured 17 stable HGSC organoids from 10 patient samples, all of which were derived from frozen specimens, with a success rate of 53%. Additionally, these organoids maintained genetic and phenotypic stability. Furthermore, the study found that there were significant differences in the drug response of organoids cultured in physiological media compared to those cultured in nutrient-rich media, suggesting that physiological media can better reflect drug responses under real physiological conditions, providing a new tool for drug screening and efficacy prediction (19).

### 3.2 Culture techniques for ovarian cancer organoids

Ovarian cancer organoid research faces a key challenge: lack of standardized culture conditions. Additionally, the construction of ovarian cancer organoids is not static but rather diverse and flexible. The key lies in the composition of the culture medium, as different medium formulations contain various growth factors, hormones, nutrients, and other components, which directly affect the efficiency and success rate of establishing organoid lines. Researchers strive to optimize growth factors, nutrients, and supplements to efficiently grow organoids. This is crucial for leveraging organoids as preclinical models for disease study, drug screening, and personalized medicine. **Table 2** shows systematic evaluation of media formulations to identify optimal components for sustained organoid expansion while maintaining fidelity to original tumors.

**Table 2:** Culture media components and success rates comparative analysis.

Study	Media Components	Success Rate
Kopper et al. (16)	Glutamax 1×, Pen/strep 0.2% (Primocin), A83-01 0.5 μM, Nicotinamide 10 mM, B27 supplement 1×, N-acetylcysteine 1.25 mM, 17-β estradiol 100 nM, EGF 5 ng/mL, FGF10 10 ng/mL, Y27632 5 μM, HEPES 10 mM	55% - 30%
Hofmann et al. (17)	Glutamax 1×, Pen/strep 100 U/ml & 100 mg/ml, Primocin 1×, Nicotinamide 1 mM, N2 supplement 1×, B27 supplement 1×, EGF 10 ng/mL, FGF10 100 ng/mL, p38i (SB203580) 0.5 μM, Noggin 100 ng/mL*, R-spo1 25%*, IGF1 20 ng/mL, Y27632 9 μM, HEPES 10 mM	44%
Maenhoudt et al. (47)	Glutamax 1×, Pen/strep 2% (Primocin), A83-01 0.25 μM, Nicotinamide 5 mM, N2 supplement 10 μM, B27 supplement 1:50, N-acetylcysteine 1.25 mM, 17-β estradiol 10 nM, EGF 50 ng/mL, FGF10 100 ng/mL, Forskolin 10 μM, Hydrocortisone 500 ng/mL, Y27632 10 μM, HEPES 10 mM	75%
Bi et al. (48)	Glutamax 1×, Primocin 1×, A83-01 0.5 μM, Nicotinamide 5 mM, B27 supplement 1×, N-acetylcysteine 1 mM, 17-β estradiol 100 nM, FGF4 10 ng/mL, Heregulin-β-1 37.5 ng/mL, EGF 5 ng/mL, FGF10 10 ng/mL	53%
Senkowski et al. (19)	Glutamax 1×, Primocin 1×, A83-01 0.5 μM, Nicotinamide 5 mM, B27 supplement 1×, N-acetylcysteine 1 mM, 17-β estradiol 100 nM, p38i (SB203580) 0.5 μM, FGF4 10 ng/mL, FGF10 10 ng/mL (Other components may vary, not fully listed)	Not Specified

Components marked with an asterisk (*) indicate that the concentration or proportion may be specific to the study and may not be directly comparable to other studies. Also, note that the Senkowski et al. (19) study does not have a fully listed set of components and success rate, so the information provided is partial and other components may vary.

Drawing upon a human FTE organoid medium as a baseline, Kopper et al. augmented this medium with hydrocortisone, forskolin, and heregulin-β-1, incorporating or excluding WNT conditioned medium, thereby establishing two distinct culture conditions. These conditions were concurrently employed to culture cells derived from a diverse range of pathological types of ovarian cancer. Following 2–3 passages, the most conducive growth medium was selected. Notably, the overall success rate of deriving organoids across all pathological types was 65%, with a notable success rate of 55% specifically for HGSOC. This approach underscores the importance of optimizing culture conditions to harness the full potential of OC organoids for research and therapeutic applications.

Maenhoudt et al. (20) optimized the culture medium for establishing organoids from HGSOC patients, and their research team successfully identified neuregulin-1 (NRG1) as a crucial factor that supports the growth and development of ovarian cancer organoids (20). Nanki et al. (21) developed a method for culturing organoids within three weeks and sequenced these organoids. They found that organoids derived from high-grade serous adenocarcinoma, clear cell carcinoma, endometrioid carcinoma, and primary tumors shared up to 59.5% of genetic variations, revealing genetic links among these different types of ovarian cancer. Notably, the researchers in this study did not add nicotinamide, neuregulin-1, cortisol, and forskolin factors to the culture medium, but instead innovatively included gastrin and insulin-like growth factor. This optimized culture medium formula may contribute to improving the success rate of culturing organoids from different subtypes of ovarian cancer, providing new tools and methods for further ovarian cancer research. Hoffmann et al (17). sampled peritoneal and ovarian primary lesions from 13 patients with advanced HGSOC (prior to chemotherapy) and successfully established 15 stable organoid lines. Their study found that activation of the Wnt signaling pathway led to growth arrest in HGSOC organoids, revealing that a low-level Wnt environment has a positive impact on the long-term stable growth of HGSOC organoids. This provides important reference for regulating the Wnt signaling pathway when culturing HGSOC organoids (17).

The lack of standardized success criteria for ovarian cancer organoid platforms hinders clinical translation due to unclear benchmarks for quality, reproducibility, and robustness. This uniformity gap impedes result comparison across labs, limiting progress. There is an urgent need for consensus standards for ovarian cancer organoid culture, which should encompass media composition, quality control measures, and standardized protocols for assessing organoid characteristics. These efforts will accelerate research and enable clinical applications, revolutionizing ovarian cancer diagnosis, treatment, and monitoring.

Personalized culturing methods are also crucial for the establishment of ovarian cancer organoids of different subtypes. Each patient’s tumor possesses unique biological characteristics, including gene expression, metabolic pathways, and drug sensitivity. By adjusting the culturing methods to target these specific characteristics, we can more accurately simulate the authentic growth environment of different subtypes of ovarian cancer *in vivo*, thereby establishing organoid models that are closer to reality and more representative. Through continuous exploration and optimization of the construction methods for ovarian cancer organoids, we hope to pave a new path for precision treatment and research of ovarian cancer. This approach not only enhances our understanding of the disease but also holds the potential to revolutionize treatment strategies, ultimately leading to improved outcomes for patients with ovarian cancer.

## 4 Organoid simulation of ovarian cancer carcinogenesis and development

Löhmussaar et al (17). successfully established organoids derived from mouse fallopian tubes and ovarian surface epithelial tissues, and further developed these organoids into tumor models aimed at exploring the development mechanisms of HGSOC. The study found that solid tumors exhibited two distinct morphologies: glandular epithelial type or mixed epithelial-mesenchymal type. Genetic expression analysis of organoids derived from different types of tumors revealed specific gene expression patterns in organoids derived from mixed-type tumors. A total of 364 genes were upregulated in these organoids, which are closely related to biological processes such as extracellular matrix organization, cell migration, cell adhesion, cell proliferation, and mesenchymal development. Subsequently, the researchers conducted a more in-depth analysis of gene expression in mixed-type organoids and found that multiple genes related to epithelial-mesenchymal transition, such as Vim, Twist1, and Zeb1/2, were upregulated in these organoids. This discovery further confirmed that mixed-type tumors possess an epithelial-mesenchymal phenotype and may enhance their invasiveness and migratory ability through the epithelial-mesenchymal transition process.

Chromosome instability, as a key factor leading to genetic and cellular phenotypic changes, has profound implications in clinical practice. Its manifestations include the generation of extrachromosomal DNA and micronuclei, the activation of innate immune signals, tumor metastasis, and the emergence of treatment resistance (22, 23). The causes of chromosome instability are extremely complex, encompassing aspects such as erroneous chromosome segregation during mitosis, defects in homologous recombination, telomere dysfunction, break-fusion-bridge cycles, and stress conditions encountered during DNA replication (24, 25). To improve the treatment outcomes of HGSOC, Maria Vias et al. constructed PDOs models that accurately reflect the patterns of chromosome instability observed in patients. They further conducted in-depth characterization analysis of the genome, transcriptome, drug sensitivity, and intratumoral heterogeneity of PDOs. Through detailed analysis of copy number features, they successfully demonstrated that these PDOs models can comprehensively recapitulate all clinically relevant genomic features of the spectrum of chromosome instability observed in HGSOC patients. The study found that regions on chromosomes 8, 10, 11, 12, 17, and 1 exhibited the most significant genomic variations. Among these variation-rich regions, researchers identified genes that were highly correlated with copy number. Notably, the MYC gene exhibited a significant correlation between copy number and gene expression, ranking first in absolute copy number in the PDOs cohort, followed by the ZWINT gene (26). These findings not only strongly confirm the effectiveness of PDOs as models for studying HGSOC tumor cells, but also provide insights into the complex relationship between gene copy number variations and gene expression. The high copy number and strong expression of genes such as MYC and ZWINT may be directly associated with the progression and malignancy of HGSOC.

Hoarau-Véchot et al. (27) developed a 3D spheroid model based on endothelial cells to more accurately simulate the *in vivo* environment. In this model, endothelial cells and ovarian cancer cells form organized vascular spheroid structures, resembling the conditions of tumors in ascites within the body. The study found that the Akt and Notch3/Jagged1 pathways play pivotal roles in the formation of vascular spheroids and peritoneal metastasis. Additionally, the research identified the roles of several important factors, such as FGF2, PTX3, PD-ECGF, and TIMP-1, in the organization of vascular spheroids. Furthermore, the Notch3/Jagged1 pathway promotes tumor proliferation and peritoneal infiltration through the interaction between ovarian cancer cells and endothelial cells. This model contributes to our understanding of the complex mechanisms of tumor metastasis, predicts the response to chemotherapy and anti-angiogenic therapy, and provides new insights for the treatment of ovarian cancer.

Ovarian cancer organoids offer a realistic *in vitro* model, reflecting genetic, morphological, and functional properties of the disease. Real-time monitoring of genetic mutations and expression changes can reveal crucial gene roles, supporting gene and targeted therapies. These organoids also serve as a tool to study cell proliferation and apoptosis, providing a basis for targeted drug development. By simulating the tumor microenvironment and exploring ovarian cancer’s interactions with it, we gain insights into its impact on cancer occurrence and progression. Many researchers are exploring this field to accurately mimic ovarian cancer’s biological behavior, aiming to comprehensively understand its pathogenesis and offer new hope for clinical treatment strategies.

## 5 Application of ovarian cancer organoids in drug screening and resistance research

Drug resistance in ovarian cancer, stemming from tumor cell alterations, poses a significant challenge to effective treatment. Understanding resistance mechanisms is crucial for the development of novel therapies. Ovarian cancer organoids, which retain tumor characteristics, serve as an invaluable tool for simulating tumor-drug interactions *in vitro*. By culturing organoids with various drugs, researchers can expose drug effects and tumor adaptations, ultimately uncovering resistance mechanisms that guide the development of targeted treatments.

### 5.1 Single-drug screening and resistance

Drug response is intimately linked to genomic features, making organoids an ideal model for drug sensitivity testing. Nanki et al. (21) examined the sensitivity of seven pairs of ovarian cancer organoids to first-line chemotherapy drugs, including cisplatin, carboplatin, and paclitaxel. Their findings revealed that BRCA1-mutated organoids were more sensitive to PARP inhibitors and platinum drugs, whereas organoids derived from clear cell carcinoma exhibited resistance to these drugs, suggesting the need for alternative treatment strategies. Similarly, Hill et al. (15) and Mengyu Tao et al. (28) conducted short-term organoid cultures, demonstrating that sensitivity to PARP inhibitors and platinum drugs is not solely determined by HRD status, with notable differences observed among patients. Gorski et al. (29) further investigated carboplatin sensitivity in HGSOC organoids, identifying genomic drivers of resistance and correlating their findings with clinical outcomes.

### 5.2 Combination drug screening resistance

Given that clinical ovarian cancer treatment often involves combination therapy, recent studies have focused on the combined drug response of organoids. De Witte et al. (30) demonstrated that combination therapy with carboplatin and paclitaxel could overcome single-drug resistance in some organoids, enhancing the clinical relevance of organoid drug sensitivity screening. Phan et al. (31)utilized a high-throughput screening platform to test the effects of 240 kinase inhibitors on ovarian cancer organoids, providing insights into the direct impact of these drugs on tumor cells.

### 5.3 Molecular mechanisms of drug resistance

To delve deeper into the molecular mechanisms underlying drug resistance, researchers have conducted RNA sequencing and functional analyses. Ziliang Wang’s team (32) discovered that FBN1 is highly expressed in cisplatin-resistant organoids, mediating chemoresistance through the VEGFR2/FAK/PKB/AKT/STAT2 pathway. They proposed a novel FBN1-targeted therapy combined with anti-angiogenic drugs. Huizhen Sun and colleagues (33) found that Aurora-A overexpression in cisplatin-resistant PDOs regulates cellular senescence and glucose metabolism via the SOX8/FOXK1 axis, contributing to chemoresistance. Gorski et al.’s *(* 29) and other studies (34) have also identified pathways associated with carboplatin resistance, such as NF-kB, cell differentiation, and PI3K-Akt signaling, as well as the role of circRAD23B in modulating drug sensitivity. The research by McCorkle and his team (35) offers new strategies for treating ovarian cancer patients resistant to paclitaxel. By establishing paclitaxel-resistant cell lines and organoid models, they discovered that lapatinib and poziotinib can inhibit the activity of ABCB1 transporter protein, enhancing the efficacy of chemotherapy drugs. Their findings suggest that combining these inhibitors with paclitaxel could synergistically inhibit tumor cell proliferation, leading to improved therapeutic outcomes.

Collectively, these studies significantly advance our understanding of drug response and resistance in ovarian cancer, highlighting the complexity and diversity of drug sensitivity. Ovarian cancer organoids effectively mimic tumor tissue changes under drug influence, providing insights into resistance mechanisms and laying the foundation for the development of novel, precision-medicine-based treatment strategies. With further research on patient-derived organoids, we anticipate the emergence of more effective personalized treatment plans that overcome drug resistance challenges, enhance therapeutic outcomes, and improve patients’ survival prognosis.

## 6 Application of ovarian cancer organoids in immunotherapy research

The lack of models that fully capture the genetic complexity and immune function of ovarian cancer hinders the evaluation of conventional, targeted, and immunotherapy approaches. To bridge this gap, researchers have explored the use of ovarian cancer organoids in immunotherapy research, leveraging their ability to mimic tumor tissue and the tumor microenvironment (TME).

### 6.1 Organoid-immune coculture approaches

To mimic the complex interplay between ovarian cancer and the immune system, researchers have adopted two primary coculture approaches. The first involves culturing tumor tissue using the air-liquid interface (ALI) method, which allows the organoids to retain endogenous immune cells such as tumor-infiltrating lymphocytes and cancer-associated fibroblasts. This approach preserves the natural immune microenvironment, enabling researchers to study its dynamics under different conditions (35, 36). The second approach involves coculturing tumor organoids with peripheral blood mononuclear cells (PBMCs) isolated from the same patient. This setup promotes the expansion of tumor-reactive immune cells and simulates the patient-specific immune response to the tumor (36).

### 6.2 Complex organoid models for immunotherapy research

Recognizing the limitations of simple organoid models in capturing the full complexity of the tumor microenvironment (TME), researchers have developed more sophisticated coculture systems. These complex organoid models incorporate not only immune cells but also stromal components such as fibroblasts (CAFs) and mesodermal progenitor cells. By coculturing organoids with PBMCs or immune cells from lymph nodes, researchers can simulate the continuous cycle of immune activation, migration, infiltration, and tumor cell elimination (37). This approach provides a more realistic model for assessing the effectiveness of immunotherapy strategies, including bispecific antibodies and CAR-NK cells (37, 38).

### 6.3 Understanding the immune microenvironment in ovarian cancer

Our current understanding of the immune microenvironment in ovarian cancer, particularly its role in driving aggressiveness and drug resistance, is still limited (39). Zhang et al. (18) made significant contributions by demonstrating that HGSOC organoids produce specific cytokines and chemokines in response to chemotherapy drugs. By blocking these chemokines, they showed that the infiltration of immune cells into the tumor microenvironment could be modulated, affecting the overall immune response. In particular, blocking CXCL10 reduced T cell numbers, while blocking GM-CSF decreased the number of tumor-associated macrophages and myeloid-derived suppressor cells (MDSCs). These findings suggest novel therapeutic strategies involving the neutralization of organoid-derived cytokines/chemokines to enhance chemotherapy efficacy or reduce its side effects (40).

### 6.4 Tumor-infiltrating lymphocytes and immune checkpoint therapy

The quality and quantity of tumor-infiltrating lymphocytes (TILs) have been shown to correlate with the clinical outcomes of ovarian cancer patients undergoing immunotherapy (18). Furthermore, tumor-infiltrating mast cells (TIMs) have emerged as important players in the immune microenvironment, exhibiting both pro-tumorigenic and immunosuppressive roles (41, 42). Using a short-term HGSOC organoid model, Cao et al. (43) validated the impact of TIMs on anti-PD1 therapy. They found that a high abundance of stromal TIMs (sTIMs) was associated with poor prognosis and the immune evasion subtype of HGSOC. In patients with low levels of sTIMs, anti-PD1 treatment was more effective in reactivating dysfunctional CD8+ T cells, as evidenced by increased proportions of GZMB+ and IFN-γ+ CD8 T cells. These findings suggest that sTIMs could serve as a biomarker for predicting response to immune checkpoint blockade (ICB) therapy and may help overcome drug resistance in HGSOC patients (44).

### 6.5 Novel strategies emerging from organoid-immune coculture research

Recent research using organoid-immune coculture systems has uncovered novel targets and mechanisms for immunotherapy. Wan et al. (44)employed a bispecific anti-PD-1/PD-L1 antibody in their HGSC organoid/immune cell co-culture system and observed significant changes in the cellular states of T cells and natural killer (NK) cells. The bispecific antibody induced NK cells to transition from an inert state to a more active and cytotoxic phenotype, suggesting that NK cells may play a previously underappreciated role in the immune response to HGSOC. Similarly, a subset of CD8 T cells transitioned from a naive state to a more active phenotype, revealing a new population of T cells responsive to ICB therapy. Further investigation revealed that these state changes were partially driven by the downregulation of the bromodomain-containing protein BRD1, suggesting that BRD1 inhibitors may enhance immunotherapy outcomes in HGSOC (44).

In conclusion, the co-culture system of ovarian cancer organoids and immune cells has emerged as a powerful tool for immunotherapy research. By simulating the complex interplay between tumor and immune cells, this approach provides novel insights into immune evasion mechanisms and facilitates the development of more effective immunotherapy strategies. With continuous improvements and optimizations, this technique is expected to play a crucial role in advancing immunotherapy for ovarian cancer patients.

## 7 Prospects and challenges of ovarian cancer organoids

The importance of personalized model systems in translational research and clinical applications is increasingly prominent. In recent years, patient-derived organoids (PDOs) have made significant progress in ovarian cancer and other fields as a model for basic and translational research. PDOs models not only facilitate our deeper understanding of the mechanisms of cancer development, but also provide strong support for the translation of laboratory research results into clinical practice. Through PDOs models, researchers can more accurately simulate the tumor growth environment and behavior within patients (45). This helps us delve deeper into genomic and transcriptomic changes, thereby determining the most suitable treatment strategies. However, despite the immense potential of PDOs models, they still face numerous challenges in practical applications. Firstly, there are significant differences in the success rates of organoid culture among different cancer types. This is mainly influenced by factors such as tumor type, pathological characteristics, and culture conditions. In particular, for specific types of cancers like epithelial ovarian cancer, the methods and conditions for organoid culture still need further optimization and improvement. Secondly, drug resistance is one of the main reasons for the failure of ovarian cancer treatment. Multiple studies have shown that PDOs models have the ability to predict clinical therapeutic responses, which can be used to investigate mechanisms related to treatment resistance and develop potential and effective treatment strategies. Nevertheless, to support their predictive value and meet the demands of clinical applications, further research is needed to obtain more accurate quantitative data and assess their applicability in different patient populations. Furthermore, PDOs models currently lack an understanding of the tumor microenvironment (TME) signature components. The TME comprises various cell types such as immune cells, stromal cells, and endothelial cells, and their interactions with tumor cells significantly impact tumor genesis, progression, and therapeutic responses. Therefore, to more accurately simulate the *in vivo* situation within patients, we need to further develop PDOs capable of modeling the tumor microenvironment and delve deeper into their interaction mechanisms with tumor cells. Cost reduction is another important factor to consider in the practical application of PDOs models. Currently, the cost of organoid culture is relatively high, limiting its widespread application in clinical and research settings. Therefore, we need to explore more economical and efficient culture methods to reduce the cost of organoid culture and expand its application scope. Despite these challenges and limitations, epithelial ovarian cancer organoids remain excellent *in vitro* platforms for preclinical studies such as drug screening, testing, early diagnostic marker discovery, DNA repair feature analysis, and recurrent organoid generation. With further research and improvements, we expect to better apply PDO models to clinical practice, providing strong support for personalized treatment and precision medicine.

## 8 Conclusions

In conclusion, PDOs have demonstrated tremendous potential and application prospects in ovarian cancer and other cancer research areas. With the continuous progress of technology and in-depth research, we are hopeful to overcome existing challenges, fully leverage the value of PDOs models in translational research and clinical applications, and provide better treatment strategies and prognoses for cancer patients.
